# Utility of Clinical, Laboratory, and Radiological Parameters for Detecting Hypovolemia in Edematous Children With Steroid-Sensitive Nephrotic Syndrome—A Prospective Observational Study

**DOI:** 10.1155/ijpe/5040795

**Published:** 2025-11-05

**Authors:** N. V. Shikha, C. Krishnan, P. K. Aslam, Gomathy Subramaniam, M. P. Jayakrishnan

**Affiliations:** ^1^Department of Pediatrics, Government Medical College, Kozhikode, India; ^2^Department of Radio Diagnosis, Government Medical College, Kozhikode, India

**Keywords:** children, hypovolemia, IVCCI, nephrotic syndrome, severe edema, SSNS, urine indices

## Abstract

A prospective observational study was conducted in 90 edematous children with steroid-sensitive nephrotic syndrome (SSNS) to determine the prevalence of hypovolemia and also to study the role of surrogate markers like severe edema, high hematocrit, inferior vena cava collapsibility index (IVCCI) ≥ 50%, blood urea/creatinine ≥ 100 : 1, and serum albumin < 1.5 gm/dL in identifying hypovolemia. The diagnostic test for hypovolemia was a combination of FeNa < 0.5 and urinary potassium (UK) index ≥ 0.6. One-third of children with SSNS had hypovolemia, of which 50% were symptomatic. High hematocrit was the most sensitive surrogate marker of hypovolemia (80%), followed by serum albumin < 1.5 g/dL (56.6%), IVCCI ≥ 50% (43%), severe edema (36.7%), and blood urea/creatinine ≥ 100 : 1 (33.3%). The specificity for detecting hypovolemia was maximum for IVCCI ≥ 50% (91.6%), followed by blood urea/creatinine ≥ 100 : 1 (90%), serum albumin < 1.5 g/dL (85%), severe edema (81.7%), and hemoconcentration (21.6%).

## 1. Introduction

Edema is the characteristic feature of nephrotic syndrome (NS). In the pathogenesis of edema, underfill due to hypoalbuminemia or overfill due to primary sodium retention can predominate on different occasions in the same patient. Whatever be the underlying mechanism, edema warrants effective therapeutic measures as, prolonged and severe edema contributes significantly to the morbidity and mortality of NS, especially in children. It is known that inadvertent use of diuretics can aggravate hypovolemia, precipitating hypotension, shock, or acute kidney injury [1] and fluid boluses or albumin infusion in a child with overfill carry the risk of precipitating pulmonary edema. All the therapeutic guidelines recommend ruling out hypovolemia before starting diuretics in NS [2].

Hypovolemia can exist without clinical manifestations in children with NS [3–5], posing a dilemma in its detection. As hypovolemia activates the renin angiotensin aldosterone system (RAAS), the high plasma renin and aldosterone levels lead to the urinary potassium (UK) excretion and sodium reabsorption. Diagnosis of hypovolemia by documenting the increased renin, Angiotensin II, and aldosterone levels is not practical in clinical settings, but in patients with urinary sodium retention (fractional excretion of sodium (FeNa) < 0.5%), a UK index ≥ 0.6 indicates those having an increased aldosterone activity in distal nephrons and thus hypovolemia [6–8]. As the UK index and other equivalent urine indices may not be uniformly available and feasible, simple and readily available clinical, hematological, biochemical, and radiological parameters can be effectively utilized to assess the volume status of children with NS. The usefulness of bedside echocardiography and bioimpedance in assessing the volume status of children with NS has been documented [9, 10]. The present study was conducted in a tertiary center from January 2018 to July 2019 to detect the prevalence of hypovolemia in children with steroid-sensitive nephrotic syndrome (SSNS) and also to determine the utility of clinical, hematological, biochemical, and radiological parameters in diagnosing hypovolemia in these children.

## 2. Methods

Children between 3 months and 12 years presenting with edema who fulfilled the criteria for relapse of NS as per the Indian Pediatric Nephrology Group guidelines 2008 were included [11]. We excluded children (i) requiring intravenous fluids or albumin infusion within 6 h of hospitalization, (ii) with relapses but not having edema, (iii) having an eGFR < 90 mL/min/1.73 m^2^ (estimated by the Schwartz formula), (iv) on fluid restriction, (v) who received diuretics or ACE inhibitors/angiotensin receptor blockers during the previous 24-h period, (vi) in whom the collection of spot urine sample within 6 h of admission was not possible, and (vii) with steroid-resistant and congenital nephrotic syndrome. All children were advised to consume a normal diet. Informed consent was taken from each parent, and institutional ethics committee approval was obtained (Ref. No. GMCKKD/RP 2018/IEC/07 dated 11.01.2018). The edema was categorized as (i) mild when there was facial puffiness and/or pedal edema involving below the level of the knee joints; (ii) moderate, when the features of mild edema were associated with ascites also; and (iii) severe, when, in addition to ascites, there was swelling extending above the knees and/or genital edema. Children were diagnosed as initial episode NS, infrequently relapsing NS (IFRNS), frequently relapsing NS (FRNS), or steroid-dependent NS (SDNS) according to the Indian Pediatric Nephrology Group guidelines [11]. They were further evaluated for invasive infections using appropriate investigations. Children detected to have peritonitis, pneumonia, cellulitis, urinary tract infections (UTIs), or septicemia were studied as a single entity named sepsis. Spontaneous bacterial peritonitis was diagnosed when children with fever, abdominal pain, tenderness, vomiting, loose stools, lethargy, or decreased appetite demonstrated >100 leukocytes/*μ*L with at least 50% neutrophils in the ascitic fluid study and/or growth of a pathogen in peritoneal fluid culture [4, 11]. Children having fever, systemic toxic symptoms, multiorgan failure, and a pathogen grown in the blood culture were diagnosed as septicemia. Children who had not passed urine for 6 h before inclusion in the study were identified as having oliguria. The clinical characteristics of hypovolemia, like lethargy, abdominal pain, tachycardia, cold extremities, low volume pulse, dehydration, and hypotension if present, were documented in all children. A spot urine sample was collected within 6 h of admission. If no urine output occurs within 6 h, the bladder was catheterized after obtaining the consent. Urine protein, sodium, potassium, and creatinine were estimated in each patient. A simultaneous blood sample was collected to estimate complete blood count, serum albumin, urea, creatinine, sodium, potassium, and cholesterol. Urine and serum electrolytes were estimated by the ion-selective electrode (ISE) method (Sensa Core's ST-100B Electrolyte analyzer). Ultrasonography examination was performed to measure the inferior vena cava diameter after resting the patients in a supine position for 5 min. Inferior vena cava diameter was measured during deep inspiration and deep expiration along the long axis below the diaphragm, 2 cm distal to the right atrium, using 3–7 or 5–18 MHz probes of the GE Voluson E8 machine in M mode. The principal investigator, who underwent multiple training sessions under a senior faculty member in the Radiology Department, performed the ultrasonography examinations.

The UK index was calculated by the formula UK^+^/(UK^+^ + UNa^+^), where UK^+^ is the urine potassium in milliequivalents per liter (mEq/L) and UNa^+^ is the urine sodium in milliequivalents per liter [12]. FeNa was calculated by the standard formula. Inferior vena cava collapsibility index (IVCCI) was calculated by the formula [(IVCe − IVCi)/IVCe] × 100, where IVCe is the maximal diameter of IVC during expiration and IVCi is the minimal diameter during inspiration [13].

Children showing both FeNa < 0.5% and UK index ≥ 0.6 were diagnosed as having hypovolemia [8, 12]. IVCCI ≥ 50%, hematocrit value ≥ 34.8% in children <5 years and ≥37.5% in children ≥5 years, blood urea (mg/dL)/creatinine (mg/dL) ratio ≥ 100 : 1, and serum albumin < 1.5 g/dL were defined as surrogate markers of hypovolemia [14, 15]. Demographic, clinical, laboratory, and radiologic characteristics of each child were documented using a structured proforma.

### 2.1. Statistical Analysis

Statistical analysis was performed by using IBM SPSS Version 18.0 (https://www.ibm.com/analytics/spss-statistics-software). Numerical variables were statistically analyzed to find the mean and standard deviation. The distribution of categorical variables was assessed by finding the frequency and proportion. The categorical outcomes were compared between the groups using the Chi-square test, and a *p* value <0.05 was considered statistically significant. Two-by-two tables were created by using a combined FeNa < 0.5% and UK index ≥ 0.6 as the reference standard for hypovolemia and IVCCI ≥ 50%, blood urea/Scr ≥ 100 : 1, serum albumin < 1.5 gm/dL, hematocrit ≥ 34.8% in children <5 years and ≥37.5% in ≥5 years, and severe edema as the index tests for hypovolemia. The sensitivity, specificity, and positive and negative predictive values of these index tests for detecting hypovolemia were calculated. Univariate analysis of clinical, radiological, and laboratory variables was carried out to find their correlation with hypovolemia, and a multivariate analysis was performed for those variables showing a positive correlation. The odds ratio and 95% confidence interval were used in the correlation studies.

## 3. Results

**Figure 1:**
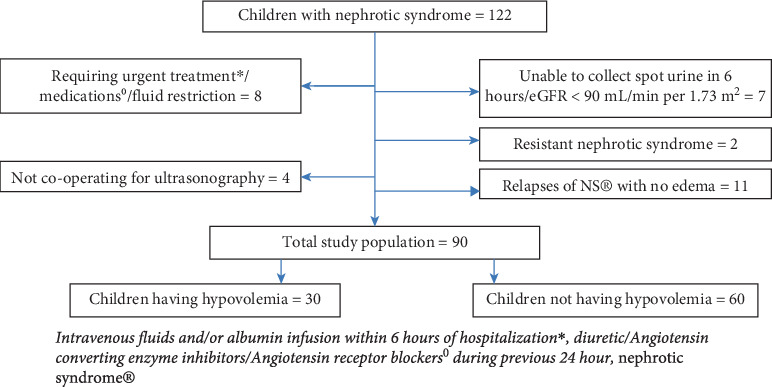
Flow chart showing selection, inclusion, exclusion, and stratification of children with nephrotic syndrome. ⁣^∗^Intravenous fluids and/or albumin infusion within 6 h of hospitalization, diuretic/angiotensin converting enzyme inhibitors/^0^angiotensin receptor blockers during the previous 24 h, and ^®^nephrotic syndrome.

Ninety edematous children with SSNS were included in the study, and hypovolemia was detected in 30 (33.4%). The selection, inclusion, exclusion, and stratification of the patients are illustrated in the flow chart (Figure 1). The demographic, clinical, laboratory, and radiological characteristics of the total study population are analyzed and illustrated in Table 1. The urine indices and IVCCI of children with NS are depicted in Table 2. The sensitivity, specificity, and positive and negative predictive values of the surrogate markers of hypovolemia are shown in Table 3 and the correlation analysis of the surrogate markers is described in Table 4. Mild, moderate, and severe edema occurred in 19 (21%), 49 (54%), and 22 (24%) children, respectively. Clinical features attributable to hypovolemia were present in 15 (50%) children with hypovolemia, which included lethargy (12.40%), abdominal pain (4.13%), tachycardia (10, 33%), cold extremities (4.13%), low volume pulse (3.10%), dehydration (4.13%), and hypotension (6.20%). Children without hypovolemia were not showing any of the clinical features. The mean serum albumin of total children, children with hypovolemia, and children with no hypovolemia is illustrated in Table 1; meanwhile, the mean serum albumin observed in children with symptomatic hypovolemia was 1.53 g/dL ± 0.46 SD. Children with severe edema and those with moderate edema lasting more than 1 week were treated with escalating doses of furosemide, thiazide, other diuretics, and albumin infusion according to the institutional protocol. Out of 22 children with severe edema, 73% required albumin infusion, 27% required furosemide infusion, and 14% required PICU admission. Edema subsided before hospital discharge in all the children. Out of 90 children with edema, 7(7.8%) had AKI at the time of presentation, and 12 (13.4%) developed AKI during the hospital course.

## 4. Discussion

In this study, we included children with SSNS having edema and studied the prevalence of hypovolemia as well as the usefulness of bedside ultrasonography and simple lab values in diagnosing hypovolemia. We used a combination of FeNa < 0.5% and UK index ≥ 0.6 as the test for detecting hypovolemia. A low FeNa indicates reduced sodium excretion, whereas a high potassium excretion index indicates increased potassium excretion, and both are due to increased aldosterone level secondary to the underlying hypovolemia [6–8]. Though FeNa is a common tool used to detect volume contraction in clinical practice, there are many limitations to this practice. The variables used to calculate the FeNa can be altered by factors like variations in the physiologic state of the body, various disease states, and the use of medications; moreover, as the FeNa represents the renal handling of solutes at a particular time only, it will not account for the dynamic nature of renal injury. Apart from volume contraction, FeNa < 1% can also occur in glomerulonephritis and interstitial nephritis. Similarly, FeNa can increase to a level of 1%–2% during a volume contracted state in situations like recent use of diuretics, adrenal insufficiency, and bicarbonaturia. In our study, the above-discussed factors affecting the FeNa have been addressed by the exclusion criteria. Our observations that, among the 57 (63.3%) children with SSNS having FeNa < 0.5%, only 30 (33%) had hypovolemia, as well as the mean FeNa in children with hypovolemia was 0.09 ± 0.08 SD (*p* < 0.01), explain the rationale of using the combination of FeNa and UK index for detecting hypovolemia.

We observed hypovolemia in one-third of children with NS during the relapses. IVCCI, severe hypoalbuminemia, and a urea/creatinine ratio > 100 were highly specific but not sensitive for detecting hypovolemia, and an elevated hematocrit showed low specificity. Severe edema as well as severe hypoalbuminemia showed a positive correlation with hypovolemia in our study.

Though symptoms and signs serve a reliable tools for suspecting hypovolemia, they alone are not enough to diagnose it, because half of the children with hypovolemia were asymptomatic in our study, which is consistent with other studies [3–6]. So screening hypovolemia by laboratory and/or radiologic parameters becomes crucial, especially before starting diuretics in children with NS.

Severe edema occurred twice commonly in hypovolemic children (*p* < 0.05), and it also showed a good specificity (81.7%); moreover, it was identified as a risk factor for hypovolemia during the correlation studies (corrected OR 3.35). Similar observations have not been documented previously to our knowledge. In the wake of the often reported clinical dilemma in identifying hypovolemia in children with severe edema, our findings on severe edema in children with NS are relevant, especially before starting diuretic therapy.

On analyzing the IVCCI data by different methods, we found that the mean IVCCI was significantly higher in children with hypovolemia compared to those without hypovolemia (*p* < 0.05), and the correlation study identified IVCCI as a strong risk factor for hypovolemia (corrected OR 20.38); moreover, it also showed high specificity (92%) to diagnose hypovolemia. With appropriate training, clinicians can use IVCCI as a bedside tool effectively to identify hypovolemia; the effectiveness of short session teaching classes in assessing the IVCCI of children has been documented [16]. Though IVCCI in our study was found unsuitable to rule out hypovolemia due to its low sensitivity (43%), it can confirm a hypovolemia suspected by other parameters (specificity 92%). Similar sensitivity and specificity for IVCCI were reported previously [10]. As young children represented a significant proportion (mean age 4.9 ± 2.46 SD) of our study population, the confounding effect of young age in the assessment of IVC collapsibility warrants further validation before its clinical application in young children.

Compared to an average serum albumin of 1.8 g/dL reported by Keenswijk et al. in hypovolemic children [7], the mean serum albumin in our study was significantly lower (1.6 g/dL ± 0.4 SD) among children with hypovolemia and even lower in symptomatic hypovolemia (1.53 g/dL ± 0.46 SD, *p* < 0.00). On further analysis, a serum albumin value of <1.5 gm/dL evolved as a risk factor for hypovolemia (corrected OR 7.3) and showed good specificity (85%). The above evidences suggest that the risk of developing hypovolemia is proportional to the severity of hypoalbuminemia. Similarly, a urea/creatinine ratio > 100 was also found useful in detecting hypovolemia in our study. The mean urea/creatinine ratio was significantly high (140.3 ± 32.4 SD) in children with hypovolemia (*p* < 0.01) and the specificity of an elevated urea/creatinine ratio was 90% for detecting hypovolemia; moreover, the urea/creatinine ratio > 100 evolved as a risk factor for hypovolemia during the multivariate analysis also (corrected OR 11.9). These findings reiterate that fluid boluses and/or albumin infusion become the priority over diuretics during the management of edematous children with NS in the context of severe hypoalbuminemia and/or disproportionately high blood urea levels.

Though a single high hematocrit value was found sensitive to detect hypovolemia (mean hematocrit value 40.7 ± 4.5 SD and sensitivity 80%), we consider this finding not sufficient for diagnosing hypovolemia owing to its low specificity (21.6%). Moreover, we used a single spot hematocrit value as the surrogate marker of hypovolemia, a recent rise of *hematocrit* ≥ 20% from the baseline being the appropriate tool for diagnosing hemoconcentration. Practicing a periodic documentation of hematocrit values in the follow up record of children with NS could overcome the clinician's practical issues of obtaining a ≥20% rise in hematocrit value at the point of care.

In our study, the male–female proportion was equal in contrast to the male preponderance reported in most of the studies. On scrutinizing the data, we found that out of 32 children excluded, 24 (75%) were girls, resulting in a lack of male predominance. FeNa < 0.5% was observed in all children with hypovolemia and 43.4% of children without hypovolemia, which goes hand in hand with the primary sodium retention occurring during the early period of relapses of children with NS.

Limitations of the study are as follows: (i) The diagnostic test for hypovolemia was not validated by using clinical criteria, (ii) predominance of young children (4.9 ± 2.46 SD) and using a single cutoff value of ≥50% IVCC instead of age- and body surface area–based cutoff values might have affected the assessment of IVCCI, (iii) a single hematocrit value instead of the rise in value was used, and (iv) urine-to-serum osmolality ratio which represent the vasopressin response in hypovolemia was not studied.

## 5. Conclusions

One-third of children with SSNS develop hypovolemia. Severe edema is commonly associated with hypovolemia, which, along with severe hypoalbuminemia, IVCCI ≥ 50%, and blood urea/creatinine ≥ 100 : 1, can be used to diagnose hypovolemia in children with NS.

## Figures and Tables

**Table 1:** Demographic, clinical, laboratory, and radiological characteristics of children with steroid-sensitive nephrotic syndrome (SSNS) (*n* = 90).

**Variables**	**Total (** **N** = 90**)**	**Hypovolemia (** **N** = 30**)**	**No hypovolemia (** **N** = 60**)**	**p** **value**
Mean age (y)	4.9 ± 2.46 SD	4.6 ± 2.42 SD	5.06 ± 2.48 SD	0.07
Male	45 (50%)	15 (50%)	30 (50%)	1.00
Female	45 (50%)	15 (50%)	30 (50%)	1.00
Initial episode	34 (37.8%)	13 (43%)	21 (35%)	0.80
Relapses	56 (61.4%)	17 (57%)	39 (65%)	0.80
Infrequent relapses	25 (27.8%)	7.0 (23.3%)	18 (30%)	0.80
Frequent relapses	8 (8.9%)	2.0 (6.7%)	6 (10%)	0.80
Steroid dependent	23 (25.6%)	8.0 (26.7%)	15 (25%)	0.80
Severe edema	22 (24.5%)	11 (36.7%)	11 (18.4%)	0.05
Sepsis	19 (21%)	10 (33%)	9 (15%)	0.04
Oliguria	7 (7.8%)	3 (10%)	4 (6%)	0.57
Hypertension	8 (8.9%)	4 (13%)	4 (6.7%)	0.30
Mean IVCCI (%)	34.8 ± 16.6 SD	44.5 ± 19.1 SD	30.0 ± 12.8 SD	0.00
Mean FeNa (%)	0.80 ± 1.2 SD	0.09 ± 0.08 SD	1.2 ± 1.3 SD	0.00
Mean urine Na (mEq/L)	57.7 ± 67.8 SD	13.4 ± 9.4 SD	79.9 ± 73.5 SD	0.00
Mean hematocrit (%)	39.7 ± 4.1 SD	40.7 ± 4.5 SD	39.3 ± 4.0 SD	0.14
Mean urea/creatinine	65.2 ± 20.1 SD	140.3 ± 32.4 SD	56.8 ± 16.6 SD	0.01
Mean albumin (g/dL)	1.8 ± 0.4 SD	1.6 ± 0.4 SD	1.9 ± 0.4 SD	0.00
Mean serum Na (mEq/L)	135.2 ± 4.8 SD	134.4 ± 4.0 SD	135.5 ± 5.3 SD	0.29

Abbreviations: FeNa, fractional excretion of sodium; IVCCI, inferior vena cava collapsibility index.

**Table 2:** Urine indices and IVCCI in children with SSNS (*n* = 90).

**Variables**		**Number (%)**
Urine sodium	<20	30 (33.3)
	>20	60 (66.7)
FeNA	<0.5%	57 (63.3)
	>0.5%	33 (36.7)
UK/UNa + UK	>0.6	30 (33.3)
	<0.6	60 (66.7)
UK/UNa + UK & FeNA	>0.6	30 (33.3)
	<0.5%	
UK/UNa + UK & FeNA	<0.6	27 (30)
	<0.5%	
UK/UNa + UK & FeNA	<0.6	33 (36.6)
	>0.5%	
IVCCI	>50%	18 (20)
	<50%	72 (80)

Abbreviations: FENA, fractional excretion of sodium; IVCCI, inferior vena cava collapsibility index (UNa in mEq/L); UNa, urine sodium.

**Table 3:** Sensitivity, specificity, and positive and negative predictive values of surrogate markers of hypovolemia in children with SSNS (*n* = 90).

**Variables**	**Sensitivity (%)**	**Specificity (%)**	**PPV (%)**	**NPV (%)**
IVCCI ≥ 50%	43.0	91.6	72.0	76.3
Severe edema	36.7	81.7	50	72.1
Hemoconcentration	80	21.6	33.8	68
Blood urea/Scr ≥ 100 : 1	33.3	90	62.5	72.9
Serum albumin < 1.5 g/dL	56.6	85	65.3	79.6

Abbreviations: IVCCI, inferior vena cava collapsibility index; NPV, negative predictive value; PPV, positive predictive value; Scr, serum creatinine.

**Table 4:** Correlation of surrogate markers with hypovolemia in children with SSNS (*N* = 90).

**Surrogate markers of hypovolemia**	**Total (** **n** = 90**)**	**Hypovolemia (** **n** = 30**)**	**No hypovolemia (** **n** = 60**)**	**p** **value**	**Odds ratio**	**95% CI**	**Corrected odds ratio**
	**Frequency (%)**	**Frequency (%)**	**Frequency (%)**				
IVCCI ≥ 50%	18 (20)	13 (43)	5 (8)	0.00	8.41	2.62–26.99	20.38
Hemoconcentration	71 (79)	24 (80)	47 (78)	0.85	1.10	0.37–3.27	1.10
Urea/Scr ≥ 100 : 1	16 (18)	10 (33)	6 (10)	0.00	4.5	1.44–13.99	11.89
Albumin < 1.5 g/dL	26 (29)	17 (57)	9 (15)	0.00	7.41	2.69–20.38	7.3
Lethargy	17 (19)	12 (40)	5 (8)	0.00	0.13	0.04–0.44	0.14
Sepsis	19 (21)	10 (33)	9 (15)	0.04	2.83	1.00–8.00	0.08
Severe edema	22 (24)	11 (37)	11 (18)	0.05	2.57	0.95–6.93	3.35

Abbreviations: IVCCI, inferior vena cava collapsibility index; Urea/Scr, blood urea/serum creatinine ratio.

## Data Availability

The data is not available publicly owing to the privacy and ethical restrictions but is available on request from the corresponding author.
